# A New Approach for Designing Fluid Concrete with Low Cement Content: Optimization of Packing Density of Aggregates

**DOI:** 10.3390/ma13184082

**Published:** 2020-09-14

**Authors:** Ning Zhang, Wenqiang Zuo, Wen Xu, Shenyou Song

**Affiliations:** 1 School of Mechanical Engineering, Southeast University, Nanjing 211189, China; nzhang_cn@seu.edu.cn; 2 Navier Laboratory, IFSTTAR/ENPC/CNRS, 77455 Champs-sur-Marne, France; 3 School of Materials Science and Engineering, Southeast University, Nanjing 211189, China; xuwen@cnjsjk.cn; 4 State Key Laboratory of High Performance Civil Engineering Materials, Nanjing 210008, China; 5 Shenzhen-Zhongshan Link Administration Center, Zhongshan 528400, China; Tonysong@163.com

**Keywords:** self-compacting concrete, packing density, strength, shrinkage, aggregate, mix proportion

## Abstract

The current study aims at proposing a novel and simple method for designing fluid concrete such as self-compacting concrete (SCC) with a low cementitious binder content to reduce the carbon footprint. Different testing methods regarding the packing density of aggregate mixtures are performed and compared. The W/C was determined according to the target compression strength. Slump flow spread is carried out to determine the most appropriate superplasticizer (SP) dosage and aggregate volume fractions and proportions in concrete mixtures. Furthermore, hardened performance, including compression strength and drying shrinkage of the fluid concrete, are characterized. Finally, a mix design process of fluid concrete with low cement content was proposed based on the preferred fresh and hardened properties of the concrete mixtures.

## 1. Introduction

Since the 1980s, construction projects have placed increasing demands on the workability of concrete. The ever-increasing complexity of the construction elements led to the difficulty of the implementation of the traditional vibrating technology, which further led to serious durability problems due to the lack of the compaction of the fresh concrete. In the meantime, the noise and interference generated during the construction of traditional vibrating concrete seriously affected the normal lives of surrounding residents. In order to solve these dilemmas, a flowable concrete, which was later named self-compacting concrete (SCC), was designed by Okamura et al. [1,2]. 

The idea behind this novel type concrete is to reduce the size and the amount of the coarse aggregates and to increase the powder and paste content in the mixture. Meanwhile, high-range water-reducing agent is required to achieve a high flowability of the interstitial cement paste while maintaining a high content of powders to provide a decent flowability for fresh fluid concrete [1,3,4,5].

Nevertheless, due to the high content of powders in SCC mix proportion, the ensuing problems are high hydration heat generated during setting and high autogenous deformation compared to the traditional vibrating concrete [6,7,8,9]. These facts could increase the stress gradient inside of the concrete elements and increase the risk of cracking and failure of the structures [6,9,10,11]. In addition, excessive use of the cementitious materials can lead to an increase of carbon footprint and be harmful to the environment [12,13]. Moreover, as the cementitious powders are the most expensive component among the SCC raw materials, the application of SCC also limited due to its high cost per unit volume [14].

In recent years, many studies have focused on reducing the amount of cementitious materials used in fluid concrete [13,15,16,17,18,19]. Three methods can be commonly used to reduce the cementitious binder content. First, the replacement of the cement clinker by grounded stone powders while maintain the same workability and mechanical performances [17,20]. Secondly, the optimization of aggregate packing density to achieve the required workability while using less interstitial paste as the lubricant phase [13,15,18]. Thirdly, a high water to powder ratio can be adopted for the structures that require low mechanical strength grade [13,14]. However, in this case, the viscosity modifying agents are usually required to maintain the consistency and to avoid the segregation of fresh mixtures.

The current study aims at proposing a novel method for designing the fluid concrete such as SCC with low cementitious binder content. Three types of packing density including the loose packing density, the vibrating density, and the compaction packing density are first carried out to determine the optimum aggregate proportioning range of binary and trinary aggregate mixtures. Slump flow spread at various age of fresh concrete mixtures containing different dosage of superplasticizer (SP) is then measured to determine the most appropriate SP dosage in concrete mixtures. After that, the fluid concrete with different aggregate volume fractions in mix proportion and fine aggregates to coarse aggregates ratio (i.e., sand ratio) are fabricated to measure the hardened performance, including compression strength and drying shrinkage. Finally, an overall design process of low cementitious binder content fluid concrete is proposed.

## 2. Materials and Methods

### 2.1. Materials

**Figure 1:**
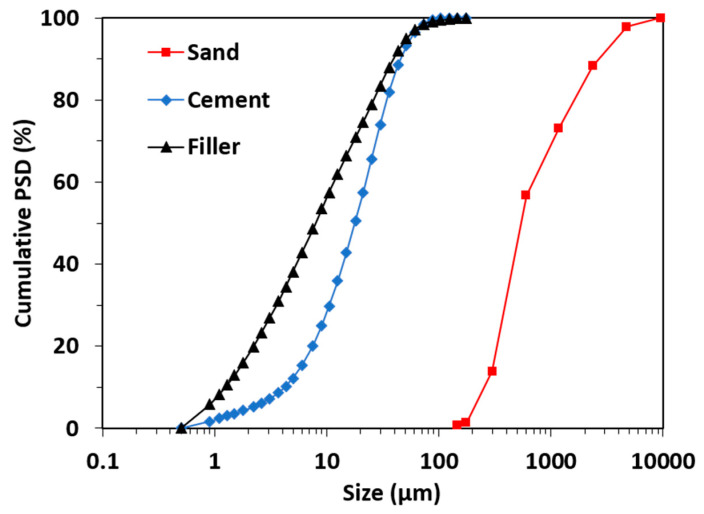
Cumulative particle size distribution of the cement powders, filler powders and sand used in this study.

A CEM I 52.5 N type of cement with apparent density of 3100 kg/m^3^ was used in this study. The filler used in this study is milled limestone with apparent density of 2700 kg/m^3^. The median particle size (i.e., the cumulated relative mass at 50%) of cement powder and filler powder are around 9 and 15 μm, respectively. River sand with apparent density of 2600 kg/m^3^ was used. The particle size distribution of cement powders, filler powders, and sand particles measured by a laser particle size analyzer are shown in Figure 1. Two sizes of crushed gravel with apparent densities of 2750 kg/m^3^ were used. The size of the coarse gravel is 5–10 mm and the size of the fine gravel is 10–20 mm.

Tap water was used as the mixing water. A polycarboxylate-type of SP with solid content of around 20% was used to adjust the flowability of the fresh concrete mixtures.

### 2.2. Testing Methods

#### 2.2.1. Packing Density of Aggregates

The binary aggregate mixture contains coarse gravels and fine gravels while the trinary aggregate mixture contains two sizes of gravels and sand, first manually mixed for 5 min to achieve a relatively homogeneous distribution.

For the loose packing density measurement, the aggregates were slowly added into a cylindric container with the volume of 7 L (both 207 mm in diameter and in height). The aggregates were slowly filled into the container with a dumping height of 50 mm to the stacked surface. Finally, the conical aggregate pile on the top of the container was gently flattened by a rod. The mass of the aggregates in the container was recorded at the end of the test.

For the vibrating packing density measurement, the container was placed on a vibrating table with the amplitude of around 1 mm and the frequency of around 50 Hz. The aggregates were slowly added to the container that is the same as the one for the loose packing density measurement. The procedures were kept the same as the loose packing density measurement. The mass of the aggregates in the container was recorded at the end of the test.

The dense packing density of aggregates was also performed using a 76-B2522 portable Gyratory Compactor (Controls Testing Equipment Ltd., London, UK). The size of the container was 150 mm in diameter. 4 kg of the manually pre-mixed aggregates were filled in the container. The shear strength ranges from 200 kPa to 600 kPa were selected to determine the effect of applied strength on the packing density of aggregates. The gyratory angle, the rotation rate, and the cycle number were set as 1.16°, 30 rpm, and 500, respectively [13,14]. The height of the aggregates in the container was recorded at the end of the test.

The packing density of aggregates mixtures performed with above three methods were computed through dividing the mass of the aggregates by the volume of the aggregates that occupied in the container. The void fraction $f_{void}$ of the aggregate mixtures can be obtained by the following formula:

(1) $$f_{void}=1-\frac{\rho_{p}}{\rho_{a}}$$

where $\rho_{p}$ is the packing density and $\rho_{a}$ the mean apparent density of aggregates, which can be estimated by the sum of the products of the volume fraction and apparent density of each type of aggregates [18].

#### 2.2.2. Mixing Procedures of Concrete Mixtures

A single-horizontal-shaft forced type of concrete mixer with mixing capacity of 60 L was used to prepare the fresh concrete mixtures. The preparation of the fresh concrete mixtures lasted in total 5 min. Dry powders and aggregates were first mixed in the mixer for 1 min. The water pre-mixed with SP was then added to the mixer and mixed for 2 min. After that, the mixer was stopped and the mixture that attached on the mixing vane was scraped in 0.5 min. Finally, the mixer was restarted for another 1.5 min.

#### 2.2.3. Slump Flow of Fresh Concrete Mixtures

Slump flow measurement was performed using a concrete slump cone according to the ASTM C1611 [21]. The flow spread was measured after the stoppage of the fresh concrete mixtures in two directions perpendicular to each other. The average of the two measured spread diameters was considered as the flow spread. For the mixtures containing different dosage of SP, three measurements, i.e., 0 h, 1 h, and 1.5 h, were performed. For the slump flow measurement at 1 h and 1.5 h, the fresh concrete mixture was stored in the mixer and was kept sealed during the resting time. The mixture was re-mixed for 1.5 min before each measurement. All the measurements were performed at the temperature of 20 ± 2 °C, and at the relative humidity of around 55 ± 5%.

#### 2.2.4. Compression Strength of Hardened Concrete

Fresh concrete mixtures were cast into the mold with the dimension of 100 × 100 × 100 mm^3^. The samples were demolded at 1 d, and then were transferred in the standard steam curing room with the temperature of 20 ± 2 °C and the relative humidity of around 98% until the testing age (3 d, 7 d, and 28 d). The compression strength of the hardened concrete samples was carried out using a universal compression machine with the maximum loading capacity of 3000 kN. The loading rate was set as 5 kN/s.

#### 2.2.5. Drying Shrinkage of Hardened Concrete

Fresh concrete mixtures were cast into the mold with the dimension of 100 × 100 × 515 mm^3^ and with an embedded copperhead in the center of one end of the mold. The samples were demolded at 1 d and then cured in the standard steam curing room at the temperature of 20 ± 2 °C and the relative humidity of around 98% for 2 days. The hardened prisms were then placed on a micrometer stand equipped with a micrometer with the measurement accuracy of ±0.001 mm. The drying deformation measurement was performed at the ambient temperature of 20 ± 2 °C and the relative humidity of around 55 ± 5%.

## 3. Experimental Results

### 3.1. Influence of Shear Strength and Cycle of the Compactor on Aggregate Packing

**Figure 2:**
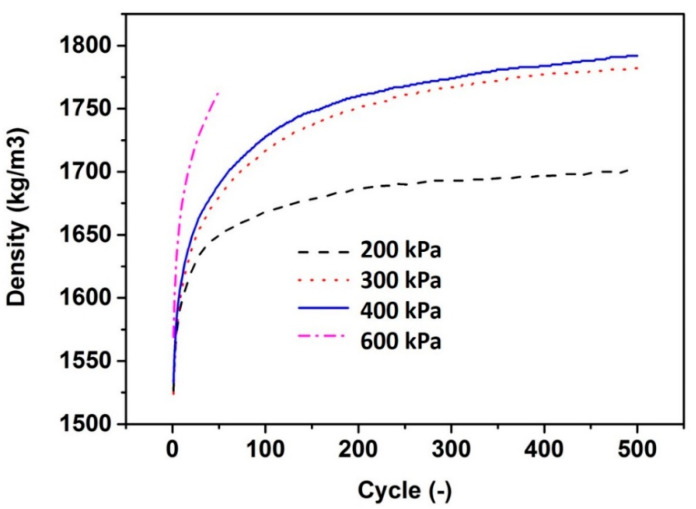
Bulk density of coarse gravels as a function of shearing cycle of the Gyratory Compactor at different shear strength.

The influence of shearing cycle on bulk density of coarse gravels measured by the Gyratory Compactor is first checked and shown in Figure 2. It can be seen that the measured density increases with the increase of applied stress at specific shearing cycles. It is moreover seen that the measured density increases rapidly at first a few tens of cycles, then it reaches plateau after around 100 cycles for the applied pressure of 200 kPa, and this value increases to around 200 for the applied pressure of both 300 kPa and 400 kPa. However, for the applied pressure of 600 kPa, the measured density shows a dramatic increase before 50 cycles. The measurement was then stopped at 50 cycles since the measured results became unrealistic due to the fact that gravel grading was largely modified under the pressure of 600 kPa.

**Figure 3:**
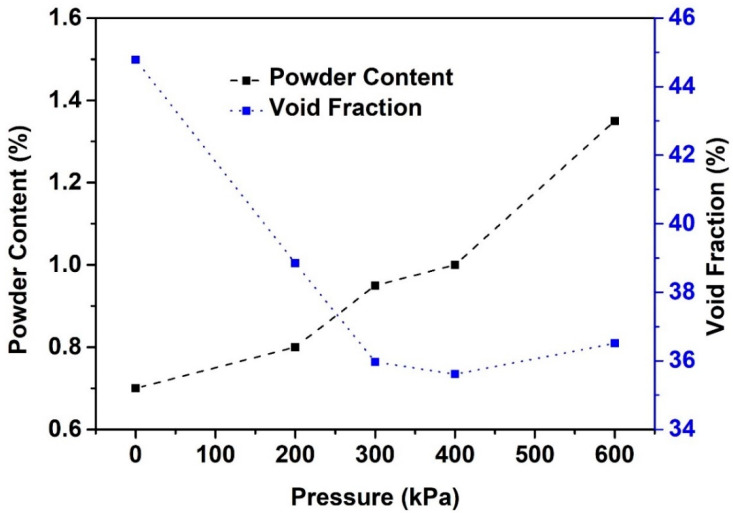
Powder content and void fraction of coarse aggregate as functions of applied shear pressure of the Gyratory Compactor. Note that for the applied pressure of 200 kPa, 300 kPa, and 400 kPa, the powder content was obtained after 500 shearing cycles; and for the applied pressure of 600 kPa, the powder content was obtained after 50 shearing cycles.

The powder content (i.e., the particle size which is smaller than 100 μm in the aggregate mixture) and void fraction of coarse gravels are plotted in Figure 3 as a function of applied pressure after certain shearing cycles. It can be clearly seen that the powder content increase with the increase of the applied pressure and the measured void fraction of coarse gravel decreases significantly with the increase of the applied pressure before 300 kPa and achieves plateau afterward. Note that the powder content after 50 cycles of 600 kPa pressure is already extremely higher than that of the 300 kPa and 400 kPa after 500 cycles. However, the corresponding void fractions remain around 36%. This means that the pressure of 600 kPa is able to significantly wear the gravel and might break the sharp corners of aggregate which can lead to a change of the particle size distribution of aggregate and thus distort the test results of bulk density. Considering the above factors, it is therefore decided to apply a pressure of 400 kPa and a cycle of 500 in the following packing density measurements of aggregate mixtures.

### 3.2. Packing Density of Binary and Trinary Aggregate Mixtures

**Figure 4:**
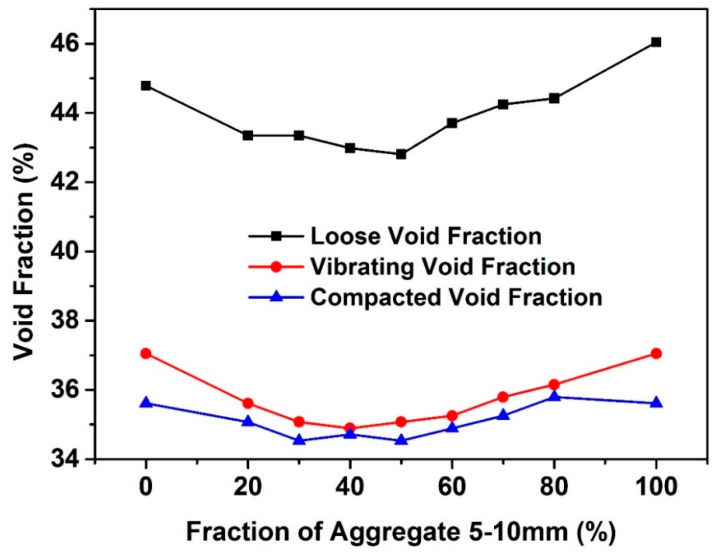
Void fraction as a function of fine gravel volume fraction in coarse-fine gravel mixtures.

Figure 4 shows the void fraction as a function of fine gravel volume fraction in coarse-fine gravel mixtures. It can be seen that the void fraction of the gravel mixtures acquired from both vibration method and compaction method shows rather similar results at each fine gravel volume fraction. The difference of the void fraction acquired by these two methods are always smaller than 2%. However, the measured loose void fraction at each fine gravel fraction is far lower than that of the vibrating void fraction and compacted void fraction. It is moreover noted that, all three methods show first a decrease then an increase of void fraction with the increase of the fine gravel volume fraction. For all the three packing methods, the lowest void fraction of the gravel mixtures achieves when the fine gravel volume fraction locates in the range of around 40–50%. This means that the relative value of the packing density of the rigid aggregate mixtures is independent of testing methods. As a matter of fact, the above results further indicate that any packing method can be utilized to identify the optimum packing density (the maximum packing density obtained from a certain packing method) of given rigid poly-dispersed particle mixtures.

**Figure 5:**
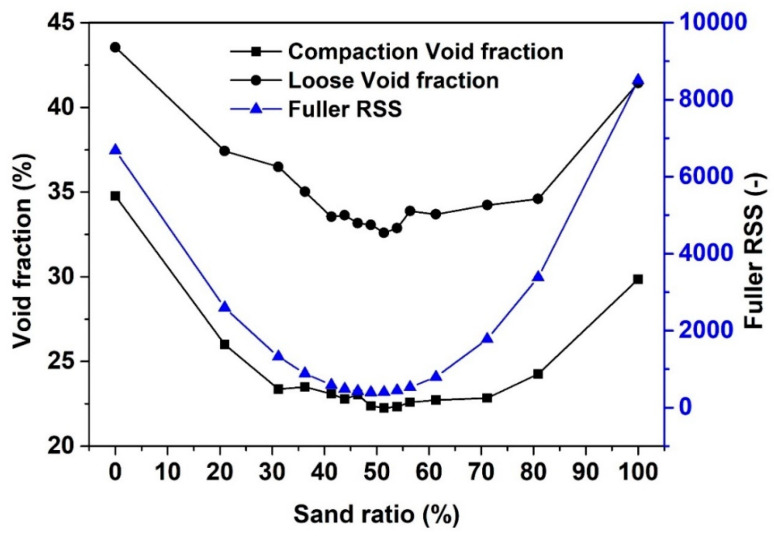
Void fraction and Fuller RSS (Cf. Equation (3)) as a function of sand volume fraction in sand-gravel mixtures.

Figure 5 shows the loose and compaction void fraction as a function of sand volume fraction in sand-gravel mixtures. Similar to the fine-coarse gravel mixtures, both the loose void fraction and compaction void fraction first decrease with the increase of sand volume fraction then increase with the increase of sand volume fraction. For both packing methods, the lowest void fraction of the sand-gravel mixtures is achieved when the sand volume fraction is around 50%. 

As a typical optimization packing model, Fuller model denotes the optimum gradation of aggregate in concrete mixture design [22]:

(2) $$P_{d}={\left(\frac{d_{i}}{d_{max}}\right)}^{0.5}$$

where $d_{i}$ is the particle size being considered, $P_{d}$ is the fraction of the particle size smaller than $d_{i}$, and $d_{max}$ is the maximum particle size in the mixture. It is proposed in [23] that the least squares method (LSM) can be adopted to obtain the optimized grading of the aggregate mixtures. This optimization algorithm aims at achieving the minimum value of the sum of the squares of the residuals (RSS, Cf. Equation (3)) at defined particle sizes between the target optimum grading and actual aggregate mixture.

(3) $$RSS=\overset{n}{\underset{i=1}{\sum }}{\left(P_{mix}\left(d_{i}\right)-P_{tar}\left(d_{i}\right)\right)}^{2}$$

where $P_{mix}$ is the composed mix, and the $P_{tar}$ is the target grading which is computed according to Equation (2). It is moreover shown in Figure 5 that the RSS between Fuller model and sand-gravel mixtures decrease with the decrease of void fraction. This indicates that the closer the grading of sand-gravel mixtures to Fuller model, the higher the packing density that the sand-gravel mixtures can achieve. 

According to the previous studies [18,24,25,26], the higher the maximum packing density of the rigid particles, the lower the yield stress of the suspensions composed by the non-colloidal particles and interstitial yield stress fluid at the same particle volume fraction (Cf. Equation (4)).

(4) $$\tau_{sus}=\tau_{i}\sqrt{\left(1-\varphi \right)/{\left(1-\varphi /\varphi_{div}\right)}^{2.5\cdot \varphi_{div}}}$$

where $\tau_{sus}$ is the yield stress of suspension composed by non-colloidal particles and interstitial yield stress fluid, which correspond to aggregates and cement paste, respectively, in the case of fluid concrete; $\tau_{i}$ is the yield stress of interstitial fluid, namely the yield stress of cement paste in concrete mixture; φ is the volume fraction of non-colloidal particles, namely the sand-gravel particles in concrete mixture; $\varphi_{div}$ is the divergence packing density of non-colloidal particles and can be expressed as $\varphi_{div}=\eta \cdot \varphi_{max}$, where $\varphi_{max}$ is the maximum packing density of non-colloidal particles and η is a coefficient varies from 0.64 to 0.88 according to [24]. Therefore, it can be deduced that by finding the optimum proportion (i.e., the maximum packing density) of existing aggregate mixtures, the minimum content of cement paste can be found to meet the required flowability or the required yield stress of concrete mixtures [27]. 

At this stage, it is proposed that the proportion of sand and gravel in concrete mixture is first determined based on the range of which the packing density of the aggregate mixtures is close to their optimum packing density. Besides, the content of the aggregate volume fraction is determined to be close to its rheology deviation volume fraction which can be estimated as 0.8 time of the dense packing density of aggregate mixtures. In the following sections, the guidelines above are always followed, in order to reduce the content of the paste and the corresponding binder content while maintaining the required flowability.

### 3.3. Determination of the Mix Proportion of Cement Paste

In order to minimize the use of cement clinker in concrete mixtures, a filler with particle size close to cement clinker is used as an alternative powder. The water to powder (W/P) value is determined according to the Abrams’ law (Cf. Equation (5)) [28,29].

(5) $$f_{c}=k_{1}\cdot \left(1/\left(W/P\right)\right)-k_{2})$$

where $f_{c}$ is the compressive strength of concrete; $k_{1}$ depends on concrete composition and degree of hydration while $k_{2}$ is a constant. In the current study, $f_{c}$, $k_{1},$ and $k_{2}$ are set as 45 MPa, 20.8, and 0.35 at the age of 28 d according to [29], respectively. It is therefore derived a W/P of around 0.39 according to the Abrams’ law.

**Figure 6:**
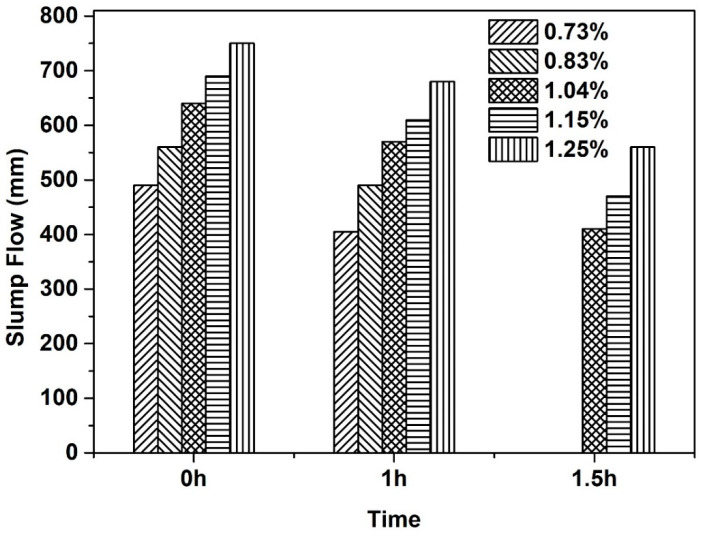
Influence of superplasticizer dosage on slump flow of fresh concrete mixtures at different time after mixing.

The impact of SP on flowability of concrete mixtures at fresh state is first studied based on one of the optimum proportions of sand-gravel mixtures. For the representative concrete mixture, the aggregate volume fraction in concrete mixture is set as 62.5%, the sand volume ratio in total aggregate mixtures is set as 53.0%. Five dosages of SP in concrete mixtures were studied and the slump flow of fresh concrete mixtures at different time after mixing is presented in Figure 6. It should be first noted that no obvious segregation phenomenon was observed for all the fresh concrete containing various SP contents and the measurement uncertainty of the slump flow in this study is always within 5% (i.e., lower than 20 mm) of the measured spread value.

It can be seen from Figure 6 that the slump flow increases with the SP dosage for all the testing ages. The initial slump flow ranges from around 500 mm to 750 mm for the concrete mixtures containing SP dosage from 0.73% to 1.25%. It also can be noted that the slump flow decreases with the increase of resting time. After a resting time of 1.0 h, all the concrete mixtures with SP dosage higher than 1.04% show the slump flow larger than 550 mm, which can be considered as SCC according to the classification of the European guidelines for SCC [30]. After a resting time of 1.5 h, the slump flow of the concrete mixtures containing SP dosage of 0.73% and 0.83% is far lower than 400 mm (the results are not shown here due to the fact that the measured value is not accurate and is less practical), and the largest slump flow (i.e. the highest SP dosage in the current study) remains at around 550 mm. It is therefore considered that the concrete mixture contains 1.04% SP as a typical fluid concrete for further optimization of the mix design in the following section on hardened properties.

### 3.4. Impact of Aggregate Content and Proportion on Fresh and Hardened Concrete

Table 1 displays the mix proportions of concrete mixtures with the sand-gravel mixtures close to their optimum proportion. It should be noted that, for all the concrete mixtures, their corresponding cement pastes always keep the same water to powder mass ratio (i.e., W/C = 0.39), the same cement to filler volume ratio (i.e., $V_{c}/V_{f}$ = 5:3), and the same SP dosage (i.e., 1.04% by mass of the powders). Two parameters are varied, namely the aggregate volume fraction and the volumetric ratio of sand to sand-gravel mixtures. The purposes are to determine the maximum aggregate content and the proper sand fraction in total aggregate mixtures that can be applied in the concrete mixture while meeting the required fresh and hardened properties.

**Figure 7:**
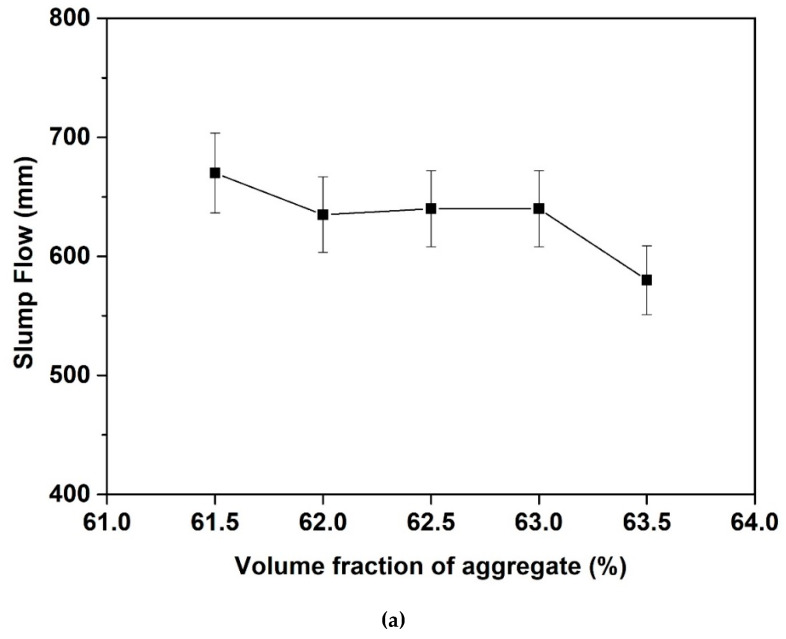

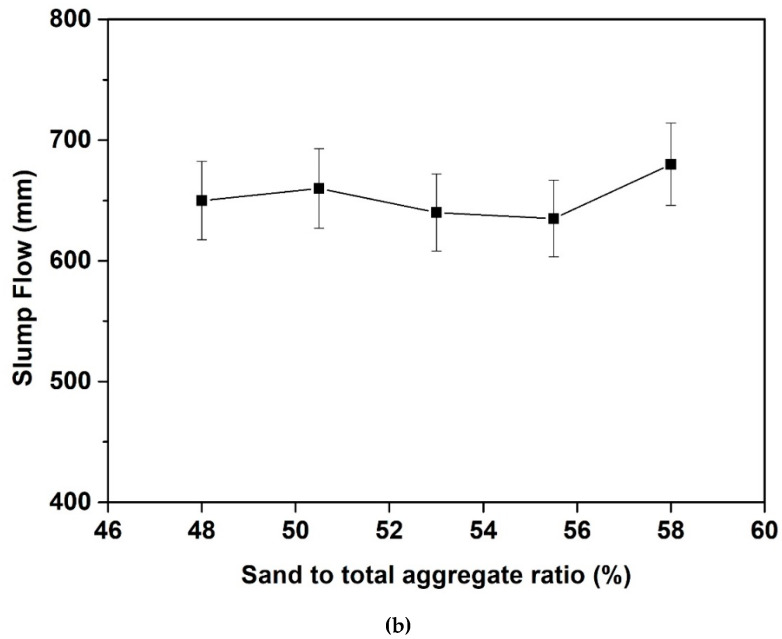
Influence of aggregate volume fraction (**a**) and sand to total aggregate volume ratio (**b**) on slump flow of fresh concrete mixtures upon mixing.

Figure 7 presents the influence of aggregate volume fraction (Cf. Figure 7a) and sand to total aggregate volume ratio (Cf. Figure 7b) on slump flow of fresh concrete mixtures upon mixing. It can be seen in Figure 7a that the slump flow decreases slightly with the increase of aggregate volume fraction as the volume fraction lower than 63%. However, an obvious drop of the slump flow is found as the aggregate volume fraction increases to 63.5%. This can be explained according to the fact that the packing fraction of aggregate mixtures achieves its rheology deviation fraction as mentioned in Equation (4). It can be seen in Figure 7b that the sand to total aggregate volume ratio has a limited impact on the slump flow of fresh concrete mixtures. This result moreover confirms that the variation of the aggregate grading (i.e., the sand to sand-gravel volume ratio in the current study) does not seem to affect the fluidity of concrete as far as the maximum packing density keeps unchanged. This is due to the fact that the flowability/yield stress of the suspensions containing yield stress interstitial fluid and non-colloidal inclusions is only dominated by the properties of the interstitial fluid (i.e., cement paste herein), the content of inclusions (i.e., aggregates herein), and its maximum packing density again according to Equation (4) and previous works [24,25,26].

**Figure 8:**
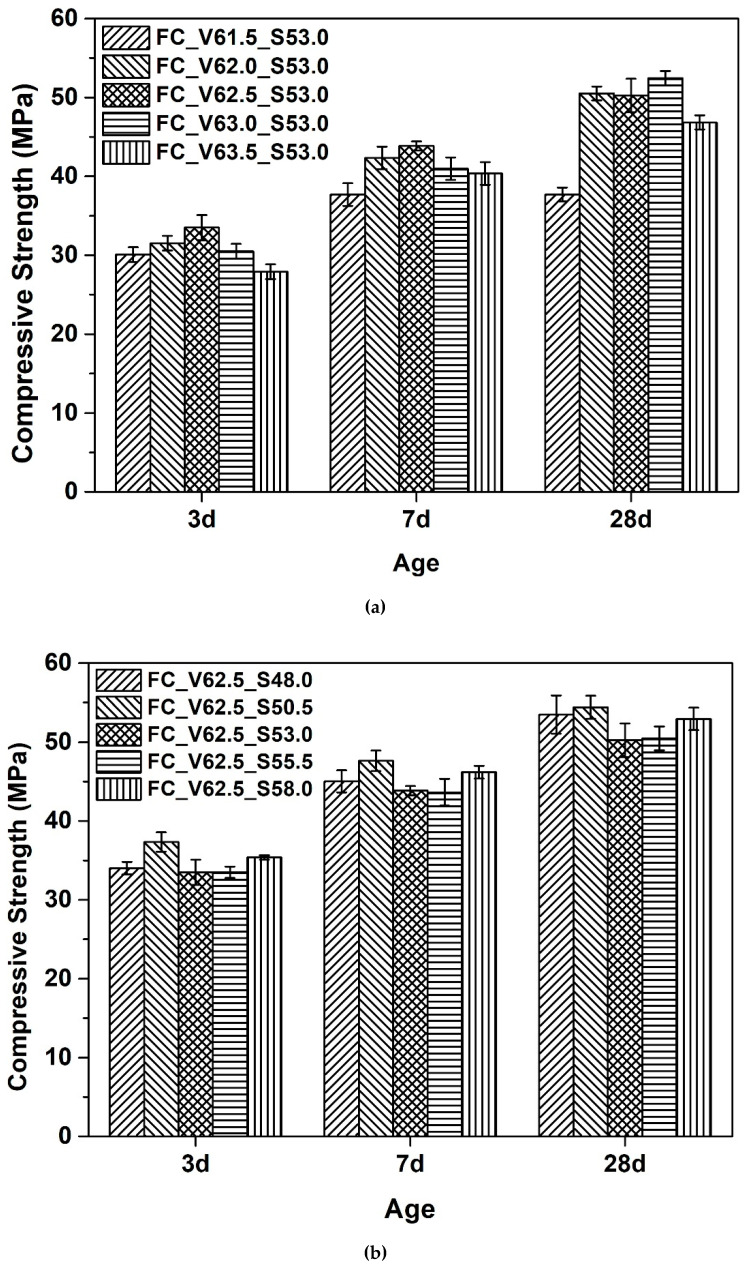
Influence of aggregate volume fraction (**a**) and sand to total aggregate volume ratio (**b**) on compressive strength of fluid concrete mixtures.

Figure 8 presents the influence of aggregate volume fraction (Cf. Figure 8a) and sand to total aggregate volume ratio (Cf. Figure 8b) on compressive strength of fluid concrete mixtures. It can be seen in Figure 8a that for the concrete mixtures containing the lowest and highest amount aggregates in the current work, the corresponding compressive strengths are markedly lower than the rest of three concrete mixtures. This phenomenon is even more obvious at later age (i.e., 28 d herein). For the concrete mixture with the lowest aggregate content, the decrease of the strength might due to the static segregation of specimen upon casting. For the concrete mixture with the highest aggregate content, the decrease of the strength can be attributed to the lack of compaction during casting since no vibration was performed.

Moreover, as seen in Figure 8b, no obvious changes in compressive strength can be found in the case of varying the sand to sand-gravel mixtures volume ratio by 10%. This can be attributed to the fact that all the concrete mixtures with different sand to sand-gravel mixtures volume ratio keep the same total aggregate content. In addition, since the concrete mixtures present similar flowability (Cf. Figure 7b) there is no obvious difference in casting process and tendency of static segregation (which can be neglected herein). It should be finally noted that, for most of the concrete mixtures with slump flow higher than 600 mm, their compressive strengths at 28 d all meet the designed strength grade (i.e., 45 MPa herein).

**Figure 9:**
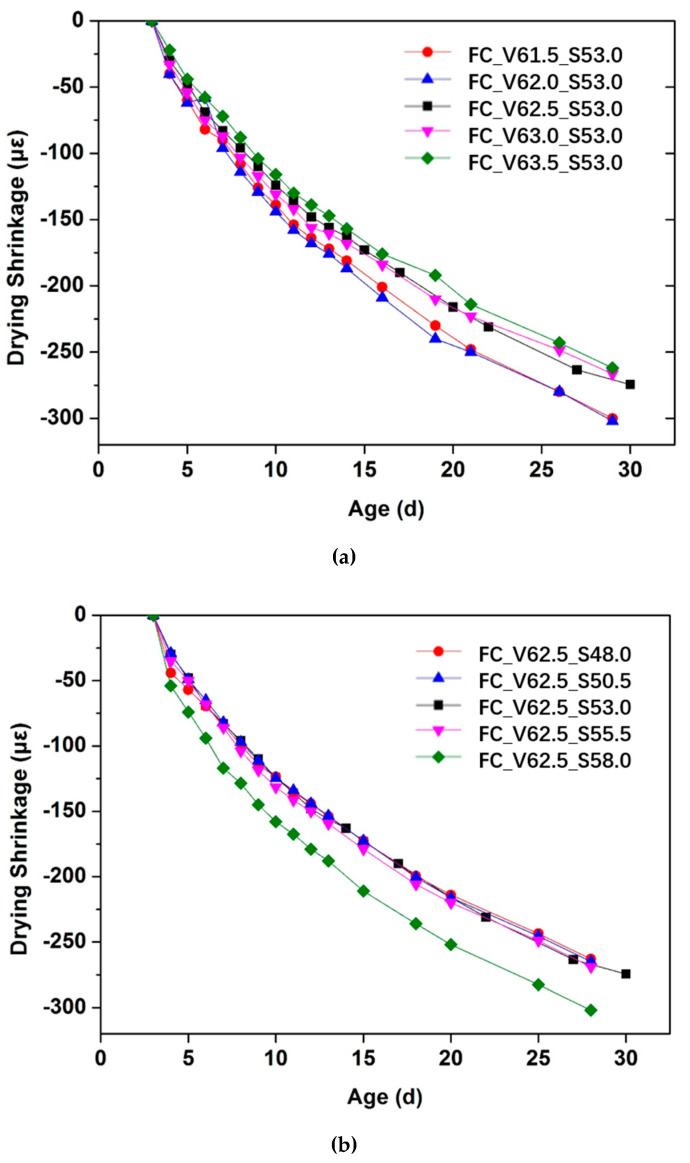
Influence of aggregate volume fraction (**a**) and sand to total aggregate volume ratio (**b**) on drying shrinkage of fluid concrete mixtures.

Figure 9 presents the influence of aggregate volume fraction (Cf. Figure 9a) and sand to total aggregate volume ratio (Cf. Figure 9b) on drying shrinkage of fluid concrete mixtures. It can be seen both in Figure 9a,b that the drying shrinkage increases rapidly during the first a couple of days and then tends to slow down. The values of the drying shrinkage for all the tested concrete mixtures at 28 d are all in the range of 250–300 micro strains. The measurement uncertainty of the drying shrinkage is around 10% of the measured value.

It can be seen in Figure 9a that the higher the aggregate volume fraction in concrete mixtures, the lower the measured drying shrinkage. This trend seems to apply to all testing ages. Since the shrinkage sources come mainly from the cement paste, for the same cement paste mix proportion, the higher the cement paste content, the larger the deformation that can be expected [11,31]. 

In the case of varying sand to total aggregate volume ratio, it is seen from Figure 9b that all the concrete mixtures with sand to total aggregate volume ratio ranging from 48.0% to 55.5% show similar evolution of drying shrinkage. However, in the case of the highest sand to total aggregate volume ratio in this study, an obvious increase of drying shrinkage can be observed compared to the ones with lower sand to total aggregate volume ratio. This can be attributed to the higher restraint effect of coarse aggregate compared to the fine aggregate, as explained in [32,33]. 

## 4. Mix Design Process and Discussion

Based on the experimental results and analysis carried out in the current study, a design process of low cement content fluid concrete (i.e., SCC) can be summarized in the following steps:

1. Determine the optimum packing of aggregate mixtures: Either the loose packing or the dense packing method can be applied to determine the range of the optimum proportion of fine aggregates (sands) and coarse aggregates (stones).
2. Determine the maximum aggregate content: Based on the dense packing density, the maximum aggregate content can be estimated according to the rheology deviation packing fraction in Chateau-Ovarlez model (Cf. Equation (4)) [25,26].
3. Determine the W/C in concrete mixtures: Based on the Abrams’ law (Cf. Equation (5)) [28,29], the W/C can be determined with a given target strength grade (cubic compression strength was proposed in the current study).
4. Determine the SP dosage: The proper dosage of SP can be acquired by a few batches of trial to meet the required flowability (other fresh properties related to workability including segregation test, filling test, and passing test, etc. [34], can be conducted to further meet the specific requirements).
5. Finalize the aggregate volume fraction and sand to total aggregate ratio: The aggregate volume fraction and sand to total aggregate ratio can be further finalized according to the mechanical properties including strength and deformation. The concrete mixtures with higher strength (mainly dominated by the flowability and segregation resistance) and lower shrinkage (lower sand to total aggregate ratio) shall be preferred.

It should be noted that, indeed, the Abrams law applied in the above mix design approach was only validated for the cement clinker [29]. However, on one hand, the limestone powder can provide sites for hydration products, and can promote the hydration process of cement minerals to a certain extent [35,36]. On the other hand, the addition of the inert filler can fill the pore space on the micron scale and can be regarded as micro aggregate which increases the mechanical performances compared to the mixture with the same W/C but without micro filler [37]. Overall, the replacement of cement clinker by limestone powder increases the W/C and will generally decrease the mechanical strength of concrete. Therefore, the consideration of limestone powder as the binder in the Abrams law can lead to an overestimation of the strength of the hardened concrete. Nevertheless, according to the mechanical strength carried out in this study, it seems that the Abrams law is able to predict the compressive strength and can be used as the strength design criteria.

In addition, in order to further guarantee the hardened properties such as the mechanical strength and shrinkage, a few more mix designs can be studied including using less filler in powder mixture or decreasing water to powder ratio to increase the hardened strength, as well as adding shrinkage-reducing admixtures to reduce the shrinkage of hardened concrete.

It is summarized that by combining the packing measurement with the Chateau-Ovarlez model, the design process of fluid concrete with paste content can be achieved. Based on existing concrete raw materials, one can efficiently locate the optimized ratio of different aggregates by simply carrying out any types of packing density measurements. The amount of aggregates that diverges the rheological behavior can also be rapidly located according to the Chateau-Ovarlez model. Finally, only a few batches are needed to verify the aggregate amount in the concrete mix proportion to meet the flowability that the fresh concrete required.

## 5. Conclusions

In the current work, a novel and simple method for designing the fluid concrete such as SCC with low cementitious binder content was carried out. Various testing methods regarding packing density were performed and compared to determine the optimum aggregate proportioning range and maximum content of aggregates in concrete mixtures. A medium strength grade concrete was designed and the W/C was determined according to the Abrams’ law. The slump flow spread at various ages of fresh concrete mixtures containing different dosages of SP was then measured to determine the most appropriate SP dosage in concrete mixtures. Furthermore, the flowability of fluid concrete with different aggregate volume fractions and sand ratios were carried out and the fresh concrete mixtures were also fabricated to measure the hardened performance, including compression strength and drying shrinkage. 

Finally, a mix design process of fluid concrete with low cement content was proposed based on the preferred fresh and hardened properties such as flowability, slump flow loss, mechanical strength, and dry shrinkage carried out in the current work. It includes the following five steps: Determine the optimum packing of aggregate mixtures; determine the maximum aggregate content; determine the W/C in concrete mixtures; determine the SP dosage; finalize the aggregate volume fraction and sand to total aggregate ratio.

## Figures and Tables

**Table 1:** Mix proportions of concrete mixtures (kg/m^3^).

Concrete Name	Cement	Filler	Water	Sand	Fine Gravel	Coarse Gravel	SP
FC_V62.5_S53.0	310	162	180	861	323	485	4.91
FC_V61.5_S53.0	319	167	185	847	318	477	5.05
FC_V62.0_S53.0	314	164	183	854	321	481	4.98
FC_V63.0_S53.0	306	160	177	868	326	489	4.84
FC_V63.5_S53.0	297	155	172	882	331	496	4.70
FC_V62.5_S48.0	310	162	180	780	358	536	4.91
FC_V62.5_S50.5	310	162	180	821	340	510	4.91
FC_V62.5_S55.5	310	162	180	902	306	459	4.91
FC_V62.5_S58.0	310	162	180	943	289	433	4.91

Note: ‘FC’ stands for fluid concrete, the second term ‘V’ stands for the volume fraction of aggregate in concrete mixtures, the last term ‘S’ stands for the sand volume fraction in sand-gravel mixtures, the numbers stand for the corresponding percentage of the items. The SP dosage is presented as its solid content which is around 20% by mass of the SP liquid.
